# Mapping Research Trends of Universal Health Coverage From 1990 to 2019: Bibliometric Analysis

**DOI:** 10.2196/24569

**Published:** 2021-01-11

**Authors:** Mahboubeh Khaton Ghanbari, Masoud Behzadifar, Leila Doshmangir, Mariano Martini, Ahad Bakhtiari, Mahtab Alikhani, Nicola Luigi Bragazzi

**Affiliations:** 1 Health Management and Economics Research Center Iran University of Medical Sciences Tehran Iran; 2 Social Determinants of Health Research Center Lorestan University of Medical Sciences Khorramabad Iran; 3 Tabriz Health Services Management Research Center Iranian Center of Excellence in Health Management Tabriz University of Medical Sciences Tabriz Iran; 4 Department of Health Sciences School of Public Health University of Genoa Genoa Italy; 5 Department of Health Management and Economics School of Public Health Tehran University of Medical Science Tehran Iran; 6 Department of Health Services Management School of Health Management and Information Sciences Iran University of Medical Sciences Tehran Iran

**Keywords:** bibliometrics, scientometrics, universal health coverage, universal health, health coverage, developing countries, low-income countries

## Abstract

**Background:**

Universal health coverage (UHC) is one of many ambitious, health-related, sustainable development goals. Sharing various experiences of achieving UHC, in terms of challenges, pitfalls, and future prospects, can help policy and decision-makers reduce the likelihood of committing errors. As such, scholarly articles and technical reports are of paramount importance in shedding light on the determinants that make it possible to achieve UHC.

**Objective:**

The purpose of this study is to conduct a comprehensive analysis of UHC-related scientific literature from 1990 to 2019.

**Methods:**

We carried out a bibliometric analysis of papers related to UHC published from January 1990 to September 2019 and indexed in Scopus via VOSviewer (version 1.6.13; CWTS). Relevant information was extracted: the number of papers published, the 20 authors with the highest number of publications in the field of UHC, the 20 journals with the highest number of publications related to UHC, the 20 most active funding sources for UHC-related research, the 20 institutes and research centers that have produced the highest number of UHC-related research papers, the 20 countries that contributed the most to the research field of UHC, the 20 most cited papers, and the latest available impact factors of journals in 2018 that included the UHC-related items under investigation.

**Results:**

In our analysis, 7224 articles were included. The publication trend was increasing, showing high interest in the scientific community. Most researchers were from the United States, the United Kingdom, and Canada, with Thailand being a notable exception. The Lancet accounted for 3.95% of published UHC-related research. Among the top 20 funding sources, the World Health Organization (WHO), the Bill and Melinda Gates Foundation, and the National Institutes of Health (NIH) accounted for 1.41%, 1.34%, and 1.02% of published UHC-related research, respectively. The highest number of citations was found for articles published in The Lancet, the American Journal of Psychiatry, and the Journal of the American Medical Association (JAMA). The top keywords were “health insurance,” “insurance,” “healthcare policy,” “healthcare delivery,” “economics,” “priority,” “healthcare cost,” “organization and management,” “health services accessibility,” “reform,” “public health,” and “health policy.”

**Conclusions:**

The findings of our study showed an increasing scholarly interest in UHC and related issues. However, most research concentrated in middle- and high-income regions and countries. Therefore, research in low-income countries should be promoted and supported, as this could enable a better understanding of the determinants of the barriers and obstacles to UHC achievement and improve global health.

## Introduction

Universal health coverage (UHC) was one of the ambitious, health-related “sustainable development goals” (SDGs) set by the United Nations (UN) General Assembly in 2015, and is one of the top priorities of their 2030 agenda. UHC represents the hope for better health for the world's poorest [1-3]. The World Health Organization (WHO) has defined UHC as a policy for “ensuring that all people can use the promotive, preventive, curative, rehabilitative and palliative health services they need, of sufficient quality to be effective, while also ensuring that the use of these services does not expose the user to financial hardship” [4].

At least half of the world's population does not have access to full coverage for a package of essential health services [5]. Health expenses lead more than 100 million people worldwide to extreme poverty every year, often forcing people to make intolerably difficult choices between purchasing food for their children and families, paying for child education, or paying for vital health services [2,6].

Countries differ in the way they address UHC provision based on a wide range of factors, such as political, economic, social, epidemiological, and technical considerations [7,8]. The path to UHC involves important policy choices and inevitable trade-offs [9]. The extent of the impact of a successful UHC implementation is referred to as the “Third Global Health Transition” [10]. Sharing various experiences of achieving UHC, in terms of challenges, pitfalls, and future prospects, can help policy and decision-makers benefit from global good practices and reduce the likelihood of committing errors and wasting resources better allocated elsewhere. As such, scholarly articles and technical reports are of paramount importance in shedding light on the determinants that make UHC achievement possible [11,12].

Nearly all of the Organization for Economic Co-operation and Development (OECD) countries and emerging economies, such as Brazil, China, Colombia, Costa Rica, India, Indonesia, and Russia, have achieved UHC [13]. These countries' experiences can be a major source of evidence of why UHC is desirable and how it should be achieved. Evidence shows a strong relationship between life expectancy at birth and UHC indicators, reflecting the 3 core dimensions of universal health coverage [14]. In moving to UHC, some countries such as Ghana, Indonesia, and Vietnam have increased their UHC indices over time, 1.43%, 1.85%, and 2.26%, respectively, mostly by improving both financial protection and service coverage [15,16].

In recent years, researchers have been using scientometrics, a branch of information science and a subfield of bibliometrics, to quantitatively investigate emerging research patterns in the scientific literature [17]. In addition, scientometrics enables an assessment of trends in article citations and how these indicators and measurements can impact policy and management. Using scholarly databases and visualization technology allows researchers to gain a good understanding of the publication trends related to a given topic [18,19].

To the best of our knowledge, there is a dearth of information concerning research patterns in the field of health care management and, specifically, UHC. Therefore, the purpose of this study is to conduct a comprehensive analysis of UHC-related scientific literature from 1990 to 2019.

## Methods

### Ethics Approval and Consent to Participate

This study was waived from ethical approval because it did not include data on animals or human subjects, and it was based on publicly available data.

### Data Sources

This quantitative study was based on medical informatics, data and text mining, and scientometrics techniques [20]. Independently, 2 authors searched Scopus from January 1, 1990, to September 24, 2019. Disagreements between them were resolved through discussion until consensus was reached.

### Inclusion and Exclusion Criteria

We limited our search to only scholarly items dealing with UHC, using “universal health coverage” as the keyword. The search was performed without language restrictions. All records relevant to the field of UHC were deemed eligible and, as such, retained in our investigation.

### Data Extraction

Data were downloaded in comma-separated values (CSV) format. Independently, 2 authors extracted relevant data, namely, (1) the number of documents published within the study period, (2) the 20 authors with the highest number of publications in the field of UHC, (3) the 20 journals with the highest number of publications related to UHC, (4) the 20 most active funding sources for UHC-related research, (5) the 20 institutes and research centers that have produced the highest number of UHC-related research papers, (6) the 20 countries that contributed the most to the research field of UHC, (7) the 20 most highly cited papers, and (8) the latest available impact factor of journals in 2018 that included the UHC-related items under investigation. Any disagreements between the 2 authors were resolved through discussion until consensus was reached.

### Data Analysis

Ad hoc visualization software was used to visualize UHC-related research hotspots, patterns, directions of research development, and other relevant trends, using networks and graphs. All data were imported and loaded into VOSviewer (version 1.6.13; CWTS). For visualization publication density worldwide (ie, publication trends among countries), the open-source tool GunnMap was used [21].

## Results

After searching Scopus, a pool of 7224 records was included in our analysis. The increasing publication trend related to UHC from January 1990 to September 2019 is shown in Table 1.

The 20 authors with the highest number of publications in the field of UHC are listed in Table 2. Of the 20 authors, 4 are from the United States, 4 are from the United Kingdom, and 3 are from Thailand.

**Figure 1:**
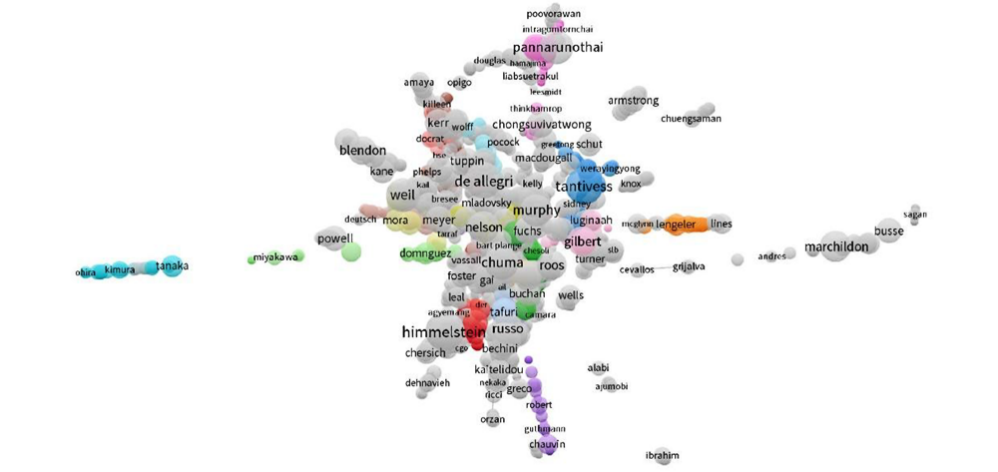
The distribution of authors publishing research in the field of universal health coverage.

The network distribution of authors publishing in the field of UHC is shown in Figure 1. The 20 journals with the highest number of publications related to UHC are listed in Table 3. *The Lancet* accounted for 3.95% of published UHC-related research.

**Table 1:** Number of publications related to universal health coverage per year, as indexed in Scopus.

Year	Number of publications
1990	25
1991	21
1992	38
1993	50
1994	103
1995	70
1996	53
1997	70
1998	77
1999	73
2000	115
2001	83
2002	87
2003	138
2004	129
2005	136
2006	188
2007	266
2008	254
2009	308
2010	251
2011	312
2012	393
2013	398
2014	449
2015	581
2016	628
2017	668
2018	784
2019	525

**Table 2:** Authors with the highest number of manuscripts related to universal health coverage.

Rank	Author’s name	Country	Number of publications	Citations	Percentage (n/7224)	H-index
1	Tangcharoensathien V	Thailand	47	3117	0.64	29
2	Atun R	United States	35	9121	0.48	48
3	Teerawattananon Y	Singapore	31	3865	0.42	27
4	Chalkidou K	United Kingdom	23	2223	0.31	22
5	McIntyre D	South Africa	23	2197	0.31	27
6	Norheim OF	Norway	23	19675	0.31	42
7	Ridde V	Canada	23	2455	0.31	24
8	Hanson K	United Kingdom	21	5268	0.28	39
9	McKee M	United Kingdom	21	51327	0.28	96
10	Mills A	United Kingdom	21	9487	0.28	56
11	Ataguba JE	South Africa	20	893	0.27	15
12	Shibuya K	Japan	20	58828	0.27	66
13	Bello AK	Canada	19	7133	0.26	32
14	Kruk ME	United States	19	4671	0.26	38
15	Woolhandler S	United States	19	10241	0.26	47
16	Limwattananon S	Thailand	18	10241	0.24	47
17	Patcharanarumol W	Thailand	18	714	0.24	12
18	Prinja S	India	18	1087	0.24	19
19	Reich MR	United States	18	3580	0.24	32
20	Bhutta ZA	Pakistan	17	69758	0.23	114

**Table 3:** Journals with the highest number of articles related to universal health coverage.

Rank	Journal	Number of publications	Percentage (n/7224)	Impact factor (2018)	Quartile in category (2018)	H-index
1	Lancet	286	3.95	59.102	Q1	700
2	Health Affairs	152	2.09	5.711	Q1	156
3	Plos One	152	2.09	2.776	Q1	268
4	BMC Health Services Research	131	1.8	1.932	Q1	90
5	Bulletin of The World Health Organization	124	1.71	6.818	Q1	148
6	Modern Healthcare	120	1.65	—^a^	Q4	9
7	International Journal for Equity in Health	115	1.58	2.473	Q1	46
8	Health Policy and Planning	105	1.44	2.717	Q1	80
9	Health Policy	95	1.31	2.075	Q1	79
10	Social Science and Medicine	93	1.28	3.087	Q1	213
11	BMC Public Health	82	1.13	2.567	Q1	117
12	Vaccine	81	1.11	4.760	Q1	164
13	Malaria Journal	77	1.06	2.798	Q1	87
14	New England Journal of Medicine	75	1.03	70.670	Q1	933
15	BMJ Global Health	63	0.86	4.28	Q1	21
16	Journal of Health Politics Policy and Law	54	0.74	1.839	Q2	48
17	American Journal of Public Health	52	0.71	0.774	Q1	236
18	Health Systems and Reform	51	0.7	—	—	—
19	International Journal of Health Planning and Management	50	0.68	1.450	Q2	37
20	Global Health Action	50	0.68	1.817	Q1	33

^a^ —not available.

Table 4 shows the 20 most active funding sources for UHC-related research. Among them, the WHO, the Bill and Melinda Gates Foundation, and the National Institutes of Health (NIH) accounted for 1.41%, 1.34%, and 1.02% of published UHC-related research, respectively.

Table 5 lists the 20 institutes and research centers that have produced the highest number of UHC-related research papers.

Table 6 shows the countries that contributed the most to the research field of UHC. Among them, the United States, the United Kingdom, and Canada contributed 2426, 919, and 545 papers, respectively.

**Figure 2:**
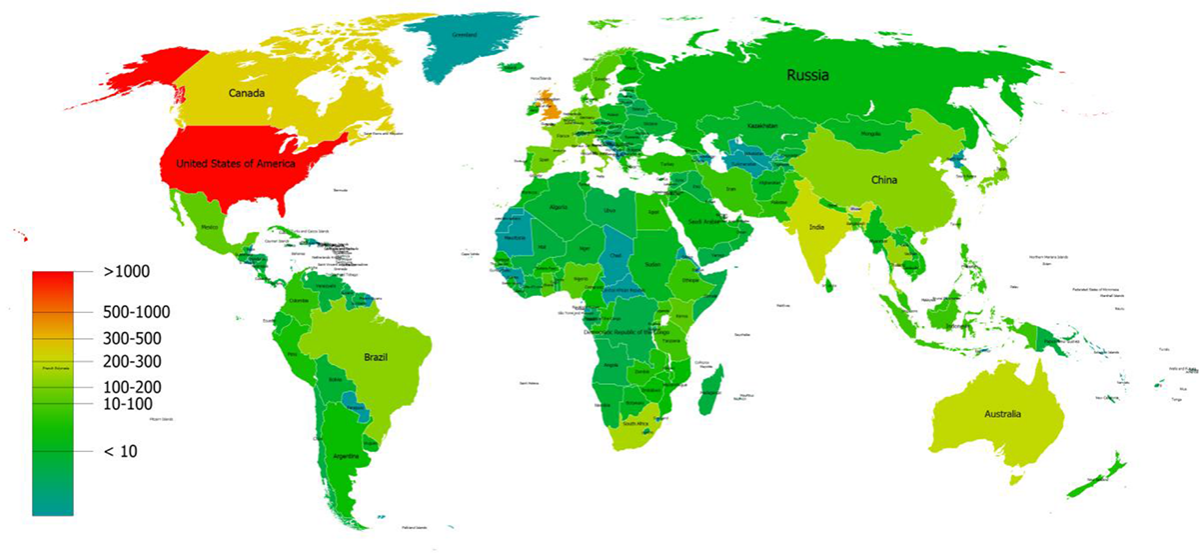
Density of publications related to the research field of universal health coverage worldwide.

Figure 2 shows the density distribution of UHC-related publications among different countries and regions around the world.

The 20 most highly cited papers are listed in Table 7. The highest number of citations was found for papers published in *The Lancet*, the *American Journal of Psychiatry*, and the *Journal of the American Medical Association* (JAMA).

**Figure 3:**
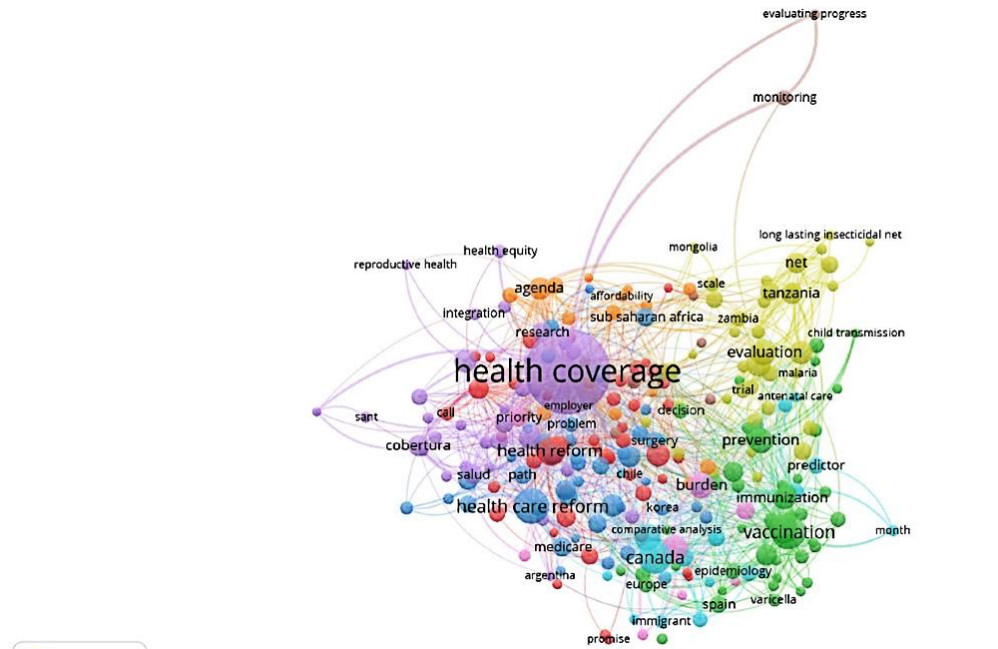
Network of the most used keywords related to universal health coverage.

In Figure 3, the network of words, themes, and topics associated with UHC is shown. Among them, the top keywords were “health insurance,” “insurance,” “healthcare policy,” “healthcare delivery,” “economics,” “priority,” “healthcare cost,” “organization and management,” “health services accessibility,” “reform,” “public health,” and “health policy.”

**Table 4:** Most active funding sources for universal health coverage (UHC)-related research.

Rank	Name of Institute	Number of publications
1	World Health Organization	102
2	London School of Hygiene & Tropical Medicine	97
3	Harvard School of Public Health	74
4	University of Toronto	65
5	Harvard Medical School	47
6	University of Cape Town	45
7	Johns Hopkins Bloomberg School of Public Health	39
8	Imperial College London	34
9	Centers for Disease Control and Prevention	33
10	Thailand Ministry of Public Health	30
11	University of California, San Francisco	26
12	Johns Hopkins University	24
13	University of Oxford	22
14	University of Washington, Seattle	22
15	University of Witwatersrand	21
16	Harvard University	21
17	Columbia University in the City of New York	20
18	The World Bank	19
19	UCL	18
20	University of Melbourne	17

**Table 5:** Highest producing institutes and research centers for universal health coverage research.

Institute	Number of publications	Percentage of total
Organisation Mondiale de la Santé	388	5.35
London School of Hygiene & Tropical Medicine	269	3.71
Harvard School of Public Health	194	2.67
University of Toronto	164	2.26
Harvard Medical School	147	2.02
University of Cape Town	112	1.54
Johns Hopkins Bloomberg School of Public Health	104	1.43
Imperial College London	104	1.43
Centers for Disease Control and Prevention	102	1.4
Thailand Ministry of Public Health	98	1.35
University of California, San Francisco	93	1.28
Johns Hopkins University	89	1.22
University of Oxford	87	1.2
University of Washington, Seattle	86	1.18
University of Witwatersrand	79	1.09
Harvard University	78	1.07
Columbia University in the City of New York	75	1.03
The World Bank, USA	73	1
UCL	69	0.95
University of Melbourne	68	0.93

**Table 6:** Countries and regions that contributed the most to the research field of universal health coverage (UHC) during 1990-2019.

Country	Number of UHC-related research papers contributed
United States	2426
United Kingdom	919
Canada	545
Switzerland	469
India	395
Australia	370
South Africa	299
Thailand	285
Brazil	219
China	215
France	205
Japan	181
Italy	176
Netherlands	173
Germany	161
Spain	158
Belgium	149
Mexico	131
Taiwan	129
Kenya	120

**Table 7:** Most cited papers related to universal health coverage.

No.	Title	Year	Journal	Number of citations
1	Evidence-based, cost-effective interventions: How many newborn babies can we save?	2005	Lancet	933
2	Social consequences of psychiatric disorders, I: Educational attainment	1995	American Journal of Psychiatry	729
3	Global Surgery 2030: Evidence and solutions for achieving health, welfare, and economic development	2015	Lancet	716
4	Socioeconomic Inequalities in Health: No Easy Solution	1993	JAMA	696
5	Hepatitis B virus infection: Epidemiology and vaccination	2006	Epidemiologic Reviews	615
6	Persistence of use of lipid-lowering medications: A cross-national study	1998	Journal of the American Medical Association	550
7	Early appraisal of China's huge and complex health-care reforms	2012	Lancet	541
8	Applying an equity lens to child health and mortality: More of the same is not enough	2003	Lancet	485
9	Taiwan's new national health insurance program: Genesis and experience so far	2003	Health Affairs	458
10	Varicella disease after introduction of varicella vaccine in the United States, 1995-2000	2002	Journal of the American Medical Association	427
11	Does universal health insurance make health care unaffordable? Lessons from Taiwan	2003	Health Affairs	406
12	Maternal and child health in Brazil: Progress and challenges	2011	Lancet	402
13	Establishment of a universal size standard strain for use with the pulsenet standardized pulsed-field gel electrophoresis protocols: Converting the national databases to the new size standard	2005	Journal of Clinical Microbiology	400
14	Help-seeking and access to mental health care in a university student population	2007	Medical Care	369
15	Policy statement: Recommendations for the prevention of pneumococcal infections, including the use of pneumococcal conjugate vaccine (Prevnar), pneumococcal polysaccharide vaccine, and antibiotic prophylaxis	2008	Pediatrics	348
16	Explaining income-related inequalities in doctor utilisation in Europe	2004	Health Economics	345
17	Access to care, health status, and health disparities in the United States and Canada: Results of a Cross-National Population-Based Survey	2006	American Journal of Public Health	325
18	The history and principles of managed competition	1993	Health Affairs	317
19	Socioeconomic Disparities in Preventive Care Persist Despite Universal Coverage: Breast and Cervical Cancer Screening in Ontario and the United States	1994	JAMA	310
20	Policy relevant determinants of health: An international perspective	2002	Health Policy	307

## Discussion

### Principal Findings

This study quantitatively assessed the publication trend related to UHC over the past 19 years. UHC-related publications have been on the rise in recent years, and this seems to be the major focus of researchers, given the important role that UHC can play in improving equity in access to health services and provisions. UHC can enable important achievements in the health sector worldwide. The growth of health-related scientific publications in the field of policy and management, and especially UHC, reflects the global interest and participation of different stakeholders, including researchers, in identifying the different dimensions and determinants that can make it possible to achieve UHC.

Undoubtedly, relying on scholarly publications can improve the performance of the health sector to achieve UHC-related goals. The rigor of the scientific method, if properly followed, can lead to fundamental changes in all areas of life, including health. Bibliometrics-based literature reviews can play an important role in examining the process of scientific publications and orienting researchers in this field [22].

From 1990 to 2019, scholarly publications in the field of UHC have been gradually increasing, especially after 2015 when policy and decision-makers have given particular emphasis to achieving UHC as one of the SDGs. Political commitment and support on this issue has contributed to the prioritization of UHC and put it on the policy and research agenda [23].

This investigation shows that authors from the United States, the United Kingdom, Canada, and Thailand produced the highest number of publications related to UHC. Scientists from the United States, the United Kingdom, and Canada have done research on the possible ways to achieve UHC goals in collaboration with various stakeholders, including health care policy and decision-makers. Thailand is one of the countries working hard to improve its health sector by making profound reforms. Since 2002, despite economic-financial problems and political instability, proper support for UHC has provided Thai citizens with a good level of health services and provisions. Therefore, researchers in this country have tried to disseminate their experiences and practices in the field of UHC to other countries around the world [24].

Usually, researchers aim to have their scientific findings published in prestigious journals so that their papers can have the highest exposure in terms of impact and receive adequate attention and citations from other researchers [25]. *The Lancet*, which has a high impact factor and plays an important role in influencing and shaping future scientific research, has published the highest number of articles related to UHC. Also, journals in the fields of health care policymaking, decision-making, and management have attracted authors' interest in submitting papers. UHC is a major topic because of its impact on all aspects of health [26].

The WHO, the London School of Hygiene and Tropical Medicine, and the Harvard School of Public Health were among the institutions and research centers that played a major role in supporting UHC-related research. The WHO's institutional nature makes it naturally interested in topics such as UHC, as it strives to provide the best evidence for a given health-related issue. The London School of Hygiene and Tropical Medicine is also one of the most prestigious institutions that, in recent decades, has promoted UHC-related studies, especially in lower-income countries, to improve health in these countries and achieve UHC goals. It strives to empower researchers in the field of health and provide high-quality public health education, as does Harvard School of Public Health.

It is important to note that, in the last decades, these two institutions have become prominent in the fields of health policy and management, indicating that they play an important role in developing UHC-related issues.

### Limitations

Despite strengths such as methodological rigor, transparency, and reproducibility, this study has some limitations that should be properly recognized. Its major limitation is the use of a single bibliographic database (Scopus). As such, results should be replicated utilizing other major scholarly databases like PubMed/MEDLINE or Web of Science.

### Conclusion

The findings of our study showed an increasing scholarly interest in UHC and related issues. However, most researchers were from the United States, the United Kingdom, and Canada, with Thailand being a notable exception. Research in low-income countries should be promoted and supported, as this could enable a better understanding of the determinants of the barriers and obstacles to UHC achievement and improve global health.

## Abbreviations

SDGs: sustainable development goals
UHC: universal health coverage
UN: United Nations
WHO: World Health Organization
